# Automatic analysis of three-dimensional cardiac tagged magnetic resonance images using neural networks trained on synthetic data

**DOI:** 10.1016/j.jocmr.2025.101869

**Published:** 2025-02-26

**Authors:** Stefano Buoso, Christian T. Stoeck, Sebastian Kozerke

**Affiliations:** a Institute for Biomedical Engineering, ETH Zurich and University Zurich, Zurich, Switzerland; b Center for Preclinical Development, University Hospital Zurich and University Zurich, Zurich, Switzerland

**Keywords:** Cardiac magnetic resonance, 3D tagged MRI, Neural networks, Cardiac motion, Strains, Image processing

## Abstract

**Background:**

Three-dimensional (3D) tagged magnetic resonance (MR) imaging enables in-vivo quantification of cardiac motion. While deep learning methods have been developed to analyze these images, they have been restricted to two-dimensional datasets. We present a deep learning approach specifically designed for displacement analysis of 3D cardiac tagged MR images.

**Methods:**

We developed two neural networks to predict left-ventricular motion throughout the cardiac cycle. Networks were trained using synthetic 3D tagged MR images, generated by combining a biophysical left-ventricular model with an analytical MR signal model. Network performance was initially validated on synthetic data, including assessment of signal-to-noise ratio sensitivity. The networks were then retrospectively evaluated on an in-vivo *external validation* human dataset and an in-vivo porcine study.

**Results:**

For the *external validation* dataset, predicted displacements deviated from manual tracking by median (interquartile range) values of 0.72 (1.17), 0.81 (1.64), and 1.12 (4.17) mm in *x*, *y*, and *z* directions, respectively. In the porcine dataset, strain measurements showed median (interquartile range) differences from manual annotations of 0.01 (0.04), 0.01 (0.06), and −0.01 (0.18) for circumferential, longitudinal, and radial components, respectively. These strain values are within physiological ranges and demonstrate superior performance of the network approach compared to existing 3D tagged image analysis methods.

**Conclusion:**

The method enables rapid analysis times of approximately 10 s per cardiac phase, making it suitable for large cohort investigations.

## Graphical abstract

**Figure:**
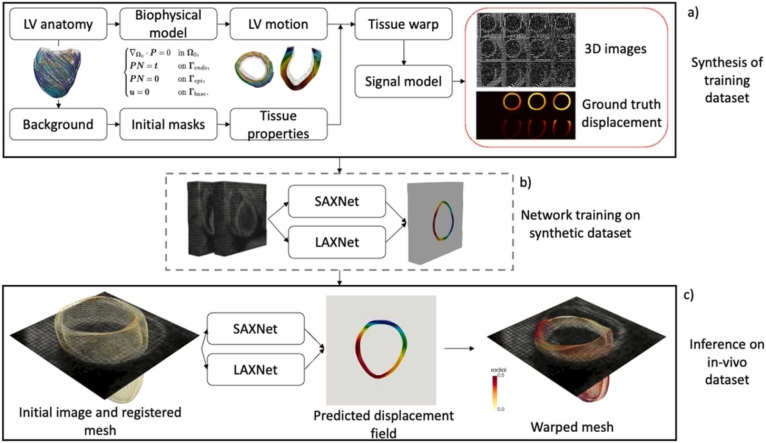

## 1 Introduction

Ejection fraction (EF), which measures cardiac chamber volume changes, remains the standard clinical metric for cardiac function assessment. However, EF has significant limitations as a diagnostic tool [1]. As a global measure, it may mask early cardiac dysfunction where impaired longitudinal contraction is compensated by increased circumferential motion. Consequently, local measurements of myocardial motion and deformation have emerged as more sensitive indicators of cardiac function [2], [3], [4].

Cardiovascular magnetic resonance (CMR) imaging enables in-vivo quantification of heart motion through three main approaches. Tagged MRI creates trackable magnetization patterns in myocardial tissue, allowing direct measurement of tissue displacement and strain [5], [6], [7]. Related to tagged MRI is displacement encoding with stimulated echoes (DENSE), which deploys an additional decoding gradient before the readout to convert tissue displacements into image phase, thereby simplifying downstream processing tasks [8], [9], [10]. Alternatively, feature tracking (FT) analyzes standard cine CMR images without requiring specialized sequences [11]. While recent neural-network-based FT methods offer rapid processing [12], they show significant inconsistencies in strain measurements compared to reference standards, particularly for radial strains [13], [11].

Tagged MRI motion analysis has traditionally relied on Fourier-based methods, image registration, and data assimilation techniques [14], [15], [16], [17]. However, these approaches are limited by lengthy processing times and significant manual intervention requirements [18].

Recent developments in convolutional neural networks have enabled automated displacement estimation from two-dimensional (2D) tagged MRI. Ferdian et al. [18] trained networks using the U.K. Imaging Biobank [19], achieving accurate circumferential and radial strain measurements compared to manual analysis. For tagged MRI, Loecher et al. [20] employed synthetic training data, demonstrating high accuracy for circumferential strains but reduced accuracy of radial strain predictions due to limited number of tag lines in the myocardium. While in-vivo training data provides realism, it requires extensive manual annotation (MA). Synthetic datasets offer known ground truth but need careful calibration of noise, signal characteristics, and resolution. Additionally, manual labeling in three-dimensional (3D) tagged MRI is inherently challenging due to resolution limitations, noise, and tag fading [21].

**Fig. 1:**
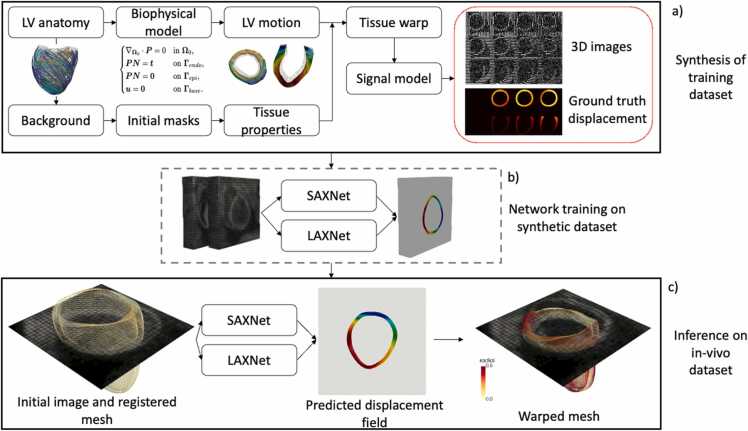
(a) Workflow for generating synthetic 3D tagged MRI: the selected LV anatomy and background define the initial tissue masks, to which tissue properties are assigned. A biophysical model generates realistic LV function and computes displacement fields to warp tissue labels in the image. The signal model produces image intensities, which, paired with ground-truth displacement values, constitute the training dataset. (b) SAXNet and LAXNet are trained on the synthetic 3D data. (c) The trained networks are applied to in-vivo data, predicting displacement fields that deform the end-diastolic reference mesh to compute displacements and strains. *SAX* short axis, *LAX* long axis, 3D three-dimensional, *LV* left ventricle, *MRI* magnetic resonance imaging.

Building on the potential of deep learning and synthetic data, we developed a method for analyzing 3D tagged MRI by leveraging deep learning and synthetic data (Fig. 1). Extending tagging to 3D enables to account for through-plane motion, which is neglected in 2D protocols.

Synthetic data were generated using a biophysical cardiac model [22] to simulate (patho)physiological left-ventricular (LV) displacements and tissue masks. Corresponding 3D tagged MRI images were synthesized with an analytical closed-form model of the complementary spatial modulation of magnetization (CSPAMM) sequence (Fig. 1a). These images were used to train two UNet-based networks, short axis netXNet) and long axis net (LAXNet), to predict displacement fields across cardiac phases relative to the end-diastolic (ED) reference frame (Fig. 1b).

The method’s displacement predictions were validated against the benchmark dataset from [17], while global metrics and physiological strains were assessed on in-vivo 3D tagged MRI from a porcine study [23], using available reference strain values (Fig. 1c). This approach advances prior neural-network-based methods by enabling automatic 3D strain analysis (radial, circumferential, and longitudinal) within anatomically aligned coordinate systems derived from cine images.

## 2 Methods

### 2.1 Synthetic image generation

**Fig. 2:**
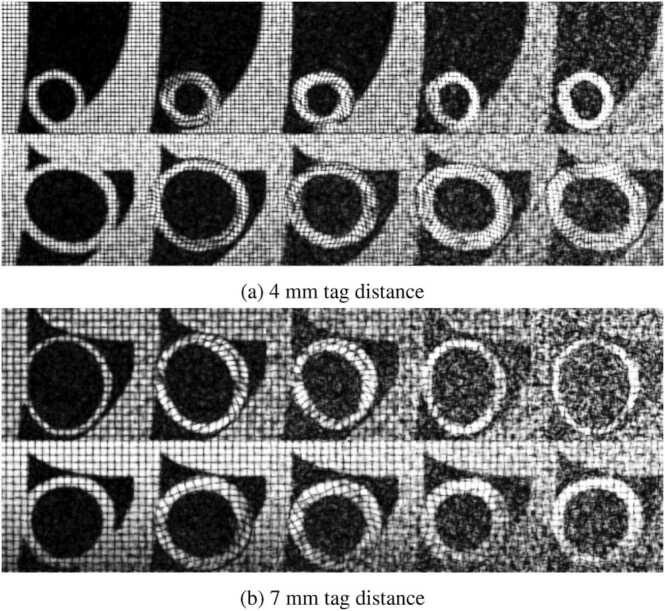
Examples of synthetic 3D tagged MRI data with 4 mm (top panel) and 7 mm (bottom panel) tag distance. Each row displays the tagged pattern on a mid-ventricular slice for selected cardiac phases. *3D* three-dimensional, *MRI* magnetic resonance imaging.

Synthetic ED LV anatomies were generated by sampling a statistical shape model described in [22]. Each anatomy was aligned along the *z*-axis of a Cartesian coordinate system to position the long-axis (LAX) direction, while the short-axis (SAX) slices were aligned with the *x* and *y* directions. The resulting shapes were projected onto a 128 × 128 × 128 grid with an isotropic resolution of 1 mm. Additional static tissue masks were added around the LV to simulate torso organs, with ellipsoids and rings representing the liver and chest (Fig. 2).

Realistic LV cardiac motion was simulated using a biophysical model based on previous work on cardiac mechanics and hyperelastic active material modeling [22], [24], [25], [26], [27]. LV tissue masks were warped according to the computed deformation fields [28]. Two synthetic MRI datasets were created by solving the Bloch equations for a CSPAMM tagging sequence, using parameters identical to those used in the in-vivo studies (Section 2.2). The signal model assumed a 1-1 tagging pulse, *t*_*aq*_ < T*_2_, and *t*_*RR*_ > T_1_, where *t*_*aq*_, T*_2_, *tRR*, and T_1_ are the acquisition time, transverse relaxation time, R-R interval, and longitudinal relaxation time, respectively. For the first dataset, a tagline distance of 7 mm was considered, while for the porcine one 4 mm was selected. Three orthogonal tag directions were simulated and successively combined, via multiplication of the complex values, after resampling to 1 mm isotropic resolution. Finally, the absolute value of the signal was computed and normalized between 0 and 1.

A total of 260 cases per dataset was simulated, with anatomical and functional parameters randomly selected as described in [22]. Specifically, nominal tissue properties were taken from [29], and uniform shear moduli determining tissue stiffness were randomly scaled in the range of [0.25–10.0]. Peak active stresses were chosen in the range of [0.1–1.0] MPa, while the prescribed diastolic filling pressures for the LV were selected in the range of [4.0–25.0] mmHg. Signal-to-noise ratio (SNR) values ranged between 5 and 35. Each simulation included 24 cardiac phases, which were stored and used for data generation. Generating the biophysical simulations and corresponding images required approximately 2 h per case.

The dataset was split into 80% for training, 10% for validation, and 10% for testing. Subsequently, patches of size 64 × 64 × 64 were extracted from each. We considered a sliding window of 16 pixels and discarded patches where less than 10% of the pixels were occupied by myocardium. This resulted in 50782, 6347, and 6347 patches for training, validation, and testing, respectively.

### 2.2 In-vivo data acquisition and processing

The in-vivo human data used in this study (referred to as *external validation* data) were sourced from the benchmark challenge dataset [17], where the acquisition parameters can be found. The 3D tagged MRI datasets were obtained with three sequential breath-hold acquisitions in each orthogonal direction (repetition time/echo time (TR/TE) = 7.0/3.2 ms, optimized sweeping flip angle, tag distance = 7 mm). Image intensities were normalized using minimum and maximum intensity values. Ground-truth landmarks were provided in the same coordinate system as the images.

**Fig. 3:**
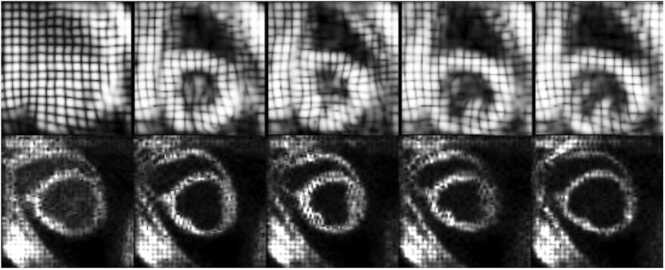
Examples of in-vivo 3D tagged MRI data for the human (top row) and porcine (bottom row) dataset. Each row displays the tagged pattern of a mid-ventricular slice for selected cardiac phases. *3D* three-dimensional, *MRI* magnetic resonance imaging.

The in-vivo porcine data consisted of 23 multi-slice cine and corresponding 3D tagged MRI datasets, retrospectively selected from a prior study [23]. Imaging was performed on a 1.5T Philips Achieva MRI system (Philips Healthcare, Best, The Netherlands) equipped with a 32-channel cardiac receiver array, following protocols approved by the Cantonal Veterinary Office (Zurich, Switzerland). Multi-slice balanced steady state free precession CMR data were acquired in the SAX orientation with a spatial resolution of 1.8 × 1.8 mm^2^, slice thickness of 8 mm, 25 cardiac phases, and TE/TR of 1.5/3 ms. 3D tagged MRI [30] utilized three CSPAMM-tagged stacks [6], with a target spatial resolution of 2 × 2 × 5 mm^3^, a field-of-view of 110 × 110 × 110 mm^3^, 14–26 cardiac phases depending on the heart rate, a tagline distance of 4 mm, TE/TR of 4.3/9.2 ms, optimized sweeping flip angle, three excitations per cardiac phase, and echo planar imaging readout with seven k-space lines per excitation. Examples of the in-vivo datasets are shown in Fig. 3.

### 2.3 Network architecture and training

**Fig. 4:**
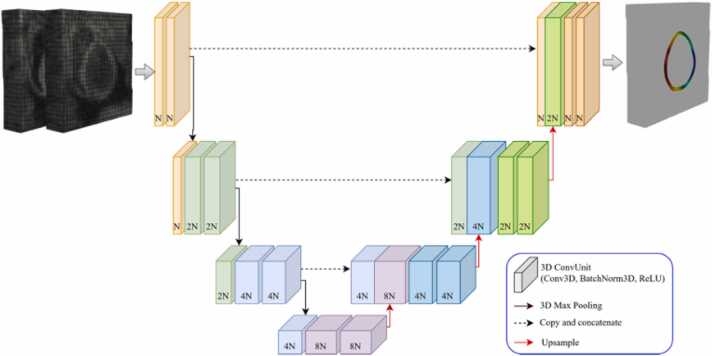
3D UNet structure: the input to the network is generated by concatenating the tagged MR images at end diastole (ED) with any other cardiac phase. The network outputs the displacement fields for the corresponding cardiac phase relative to ED. The SAXNet is used to predict displacements in the short-axis plane (with a two-channel output), while the LAXNet independently predicts the displacements in the long-axis direction (with a one-channel output). In the figure, N represents the reference number of channels, which is doubled at each step in the down-sampling branch of the UNet. For both SAXNet and LAXNet, N = 64. The network structure is available from https://github.com/yhygao/CBIM-Medical-Image-Segmentation. *3D* three-dimensional, *ReLU* rectified linear units.

For each of the in-vivo datasets, two separate networks, SAXNet and LAXNet, were trained to infer the displacements in the SAX and LAX views, respectively. The networks were based on the 3D UNet architecture (shown in Fig. 4), using implementations from [31], [32]. The network inputs were generated by concatenating the tagged MRI image at ED (the first phase acquired) with any other phase of the cardiac cycle. The network outputs were the displacement fields relative to ED for the SAX and LAX views. Training was performed for 25 epochs using the Adam optimizer [33] with a mean squared error loss function computed only in the myocardium, batch size of 32, and an initial learning rate of 1 × 10^−4^, which was halved up to three times during training, if the loss on the validation dataset worsened. Training each network required approximately 70 h on an Nvidia Titan RTX. Inference times were about 10 s per cardiac phase.

## 3 Analysis

For the synthetic data, 3D displacements were estimated using the two networks and projected onto the ED LV mesh for all frames, assuming that the real anatomy was known from the ground truth. These predictions were then compared with the ground-truth values provided by the biophysical model. Radial strains, *e*_*r*_, were computed as in [22]. The Green-Lagrangian strain tensor, computed from the inferred displacement field, was projected onto the local radial direction of the left ventricle and averaged across the entire anatomy. Circumferential, *e*_*c*_, and longitudinal, *e*_*l*_, were computed from the masks obtained from the ED meshes, which were warped with the inferred displacement fields. Circumferential strains were derived from the average diameters of the mid-wall masks in SAX views [34], while longitudinal strains were computed from the length of the skeletons of the masks in two LAX views. This approach was chosen to align with the method used in the manual analysis of in-vivo data used as reference.

For all synthetic analyses, the absolute value of the displacement error, Δ_*x*∕*y*∕*z*_, and strain errors, Δ_*r*∕*l*∕*c*_, were computed relative to the ground-truth values. All error distributions are presented as M(Q1/Q3).

The impact of SNR on networks' performance was investigated by selecting 10 simulated cases and by generating the resulting synthetic tagged MRI data for SNR values of [5, 15, 25, 35].

For the in-vivo human data, selected landmarks points tracked over the cardiac cycle were available. Therefore, the landmarks at the reference cardiac phase were warped using the displacement fields predicted by the networks. The absolute value of the displacement error, Δ_*x*∕*y*∕*z*_ was computed over time against the ground-truth values. All error distributions are presented as M(Q1/Q3) where M, Q1, and Q3 are the median, lower, and upper quartiles, respectively.

For the in-vivo porcine data, ground-truth anatomical shapes were not available. To derive the anatomical model for strain computation, parameterized anatomies were automatically fitted to the ED phase of the CMR cine data using the method presented in [35]. The ED meshes were aligned to the corresponding tagged MRI using the digital imaging and communications in medicine header information to account for the difference in geometries, off-centers, and angulation between the two scans. Next, 3D displacements were estimated using the trained networks, and peak-systolic (PS) strains, $e_{r}^{PS},e_{c}^{PS},e_{l}^{PS}$, were computed. Strain values were compared with those obtained from MA on the corresponding cine data from [23], using the semi-automatic software Segment CMR (Medviso, Lund, Sweden). All steps of our 3D tagged CMR analysis were fully automatic, with no user intervention required.

## 4 Results

### 4.1 Synthetic data analysis

**Fig. 5:**
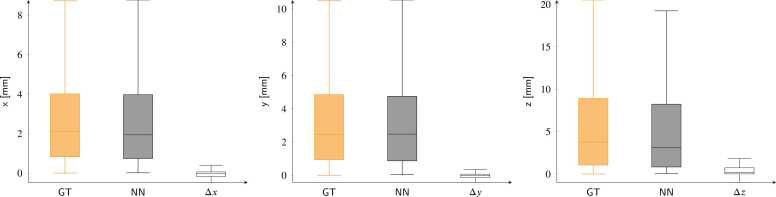
Predicted displacements and errors for all cardiac phases in the synthetic test cases are shown for the *x*, *y*, and *z* components (left, middle, and right). The orange, gray, and empty box plots represent the ground truth (GT), network predictions (NN), and absolute errors (Δ_*x*,*y*,*z*_), respectively. Pearson correlation coefficients between ground truth and predicted displacement distributions are 0.98, 0.96, and 0.93 for the *x*, *y*, and *z* components, respectively.

Displacement inference results on synthetic test data are shown in Fig. 5 collectively for all phases of the cardiac cycles. Median values and quartiles for ground-truth displacement values, in mm, are 2.11 (0.82/4.00), 2.46 (0.93/4.84), and 3.76 (1.05/8.91) for the three spatial components (*x*, *y*, and *z*), respectively. Predicted values from the networks, in mm, are 1.93 (0.73/3.97), 2.47 (0.86/4.75), and 3.14 (0.82/8.23), respectively. The resulting errors, in mm, are −0.04 (−0.16/0.05), −0.01 (−0.14/0.06), and 0.16 (0.01/0.73), respectively.

**Fig. 6:**
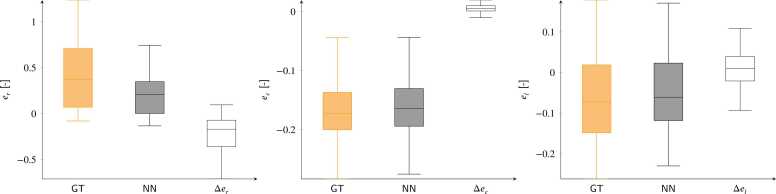
Predicted strains and errors for the synthetic test cases are shown for the radial (*e*_*r*_), circumferential (*e*_*c*_), and longitudinal (*e*_*l*_) directions (left, middle, and right). The orange, gray, and empty box plots represent the ground truth (GT), network predictions (NN), and absolute errors ($\Delta_{e_{r},e_{c},e_{l}}$), respectively. Pearson correlation coefficients between ground truth and predicted strain distributions are 0.96, 0.74, and 0.99 for *e*_*r*_, *e*_*c*_, and *e*_*l*_, respectively.

Strain value analysis is shown in Fig. 6. Median and quartiles ground-truth values are 0.37 (0.07/0.71), −0.17 (−0.20/−0.13) and −0.07 (−0.15/0.02) for *e*_*r*_, *e*_*c*_, and *e*_*l*_, respectively. Values derived from networks predictions are 0.21 (0.01/0.35), −0.16 (−0.19/−0.13), and −0.06 (−0.12/0.02), respectively. The resulting absolute error distributions are −0.16 (−0.35/−0.07), 0.00 (−0.01/0.00), and 0.01 (−0.02/0.04), respectively.

**Fig. 7:**
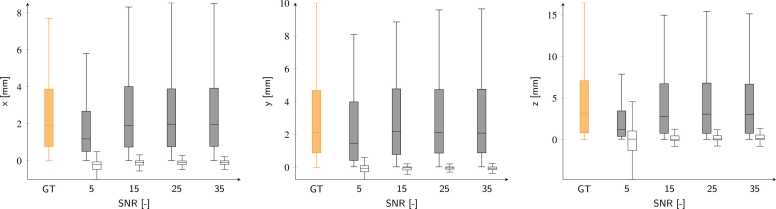
Predicted displacement and errors, for all cardiac phases, for the synthetic test cases for the *x*, *y*, and *z* components as a function of SNR. Orange boxes refer to ground-truth values (GT), while gray and empty box plots refer to network predictions and absolute errors, respectively. *SNR* signal-to-noise ratio.

**Fig. 8:**
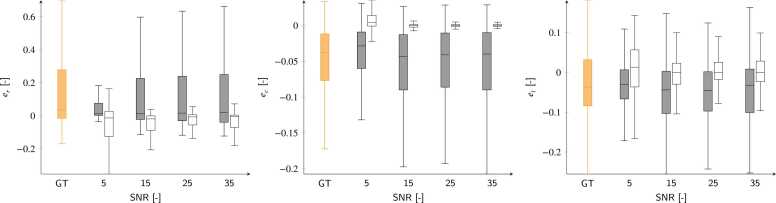
Predicted displacement and errors for the synthetic test cases for strain components as a function of SNR. Orange boxes refer to ground-truth values (GT), while gray and empty box plots refer to network predictions and absolute errors, respectively. *SNR* signal-to-noise ratio.

The effect of SNR on inference performance is shown in Figs. 7 and 8 for displacements and strains, respectively. Error values are reported in Tables 1 and 2 for displacements and strains, respectively.

**Table 1:** Displacement prediction errors for the synthetic SNR study shown in Fig. 7 (empty boxes).

SNR	Δ*x* [mm]	Δ*y* [mm]	Δ*z* [mm]
5	−0.20 (−0.47/−0.06)	−0.04 (−0.27/0.10)	0.07 (−1.30/1.04)
15	−0.09 (−0.23/0.00)	−0.03 (−0.15/0.03)	0.06 (−0.09/0.45)
25	−0.09 (−0.19/0.00)	−0.03 (−0.10/0.03)	0.08 (−0.02/0.46)
35	−0.09 (−0.19/0.00)	−0.04 (−0.13/0.03)	0.13 (−0.02/0.50)

Errors are presented as M(Q1/Q3).

*SNR* signal-to-noise ratio

**Table 2:** Strain prediction errors for the synthetic SNR study shown in Fig. 8 (empty boxes).

SNR	$\Delta_{e_{r}}$ [-]	$\Delta_{e_{c}}$ [-]	$\Delta_{e_{l}}$ [-]
5	−0.013 (−0.126/0.027)	0.004 (0.001/0.014)	0.013 (−0.036/0.056)
15	−0.019 (−0.089/−0.001)	−0.002 (−0.002/0.001)	−0.001 (−0.030/0.023)
25	−0.007 (−0.056/0.001)	−0.003 (−0.002/0.001)	0.000 (−0.017/0.025)
35	−0.006 (−0.060/0.003)	−0.001 (−0.001/0.001)	0.000 (−0.023/0.028)

Errors are presented as M(Q1/Q3).

*SNR* signal-to-noise ratio

### 4.2 In-vivo data analysis

**Fig. 9:**
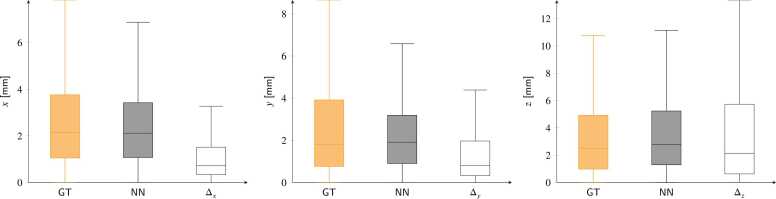
Predicted displacements for the *x*, *y*, and *z* directions on the *external validation* dataset and comparison with values derived from manual landmark tracking. Orange, gray, and empty box plots refer to manually tracked landmarks (GT), network predictions (NN), and absolute errors (Δ_*x*∕*y*∕*z*_), respectively. Pearson correlation coefficients between ground truth and predicted displacement distributions are 0.72, 0.77, and 0.50 for *x*, *y*, and *z* components, respectively.

Fig. 9 shows displacements in the *x*, *y*, and *z* directions computed from manually tracked landmarks and predictions from neural networks in the *external validation* dataset. Reference displacement values, in mm, are 2.16 (1.05/3.76), 1.82 (0.76/3.92), and 2.50 (0.98/4.92) for *x*, *y*, and *z*, respectively. From our predictions, displacement values, in mm, are 2.11 (1.07/3.40), 1.91 (0.90/3.19), and 2.10 (0.39/0.72) for *x*, *y*, and *z*, respectively. Absolute errors are 0.72 (0.34/1.51), 0.81 (0.33/1.97), and 1.12 (0.39/4.56) for *x*, *y*, and *z*, respectively.

**Fig. 10:**
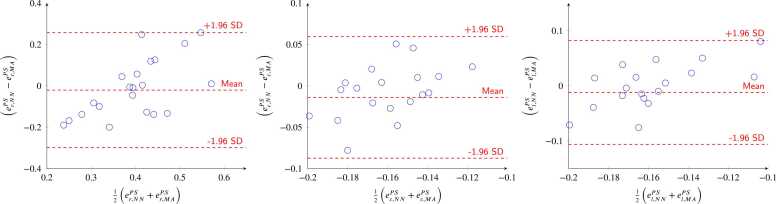
Bland-Altman plots of peak-systolic strains for radial ($e_{r}^{PS}$), circumferential ($e_{c}^{PS}$), and longitudinal ($e_{l}^{PS}$) directions of the in-vivo porcine dataset. The comparison is between values derived from manual annotations (MA) [23] and those from the neural network predictions (NN). *SD* standard deviation.

Fig. 10 shows Bland-Altman plots for PS strains from MAs on cine data and those computed from the 3D displacement fields inferred by the networks on the in-vivo porcine dataset. PS strain values of MA are 0.39 (0.35/0.42), −0.16 (−0.18/−0.15) and −0.15 (−0.20/−0.14) for $e_{r}^{PS},e_{c}^{PS}$, and $e_{l}^{PS}$, respectively. From our predictions, strain values are 0.39 (0.27/0.49), −0.17 (−0.19/−0.14), and −0.17 (−0.20/−0.15) for $e_{r}^{PS},e_{c}^{PS}$, and $e_{l}^{PS}$, respectively. Absolute errors are −0.01 (−0.13/0.05), 0.01(−0.03/0.01), and 0.01(−0.04/0.02) for $e_{r}^{PS},e_{c}^{PS}$, and $e_{l}^{PS}$, respectively.

**Fig. 11:**
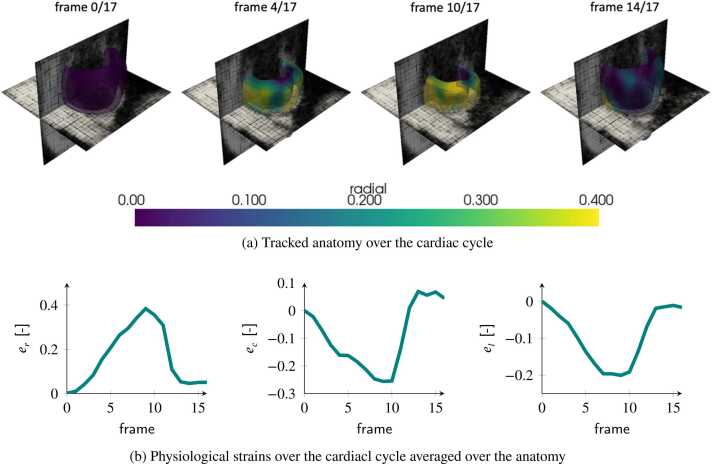
Strain analysis on the case of the in-vivo dataset. (a) Tracked anatomy superimposed onto the tagged images and (b) anatomy-averaged strains over time

Fig. 11 shows the LV dynamics over the cardiac cycle for one in-vivo case. Fig. 11a plots the overlay of the mesh model onto the 3D tagged MRI. The colorbar refers to the local radial strain computed from the inferred displacement field. Fig. 11b presents the evolution of strains throughout the cardiac cycle.

## 5 Discussion

We have presented a neural-network-based method exclusively trained on synthetic data for the automatic calculation of displacements and strains from 3D tagged MRI. Displacement tracking errors on synthetic data showed subvoxel accuracy, with interquartile error ranges better than the MRI spatial resolution. These errors (Fig. 5) were consistent with those reported in previous studies, where median error values ranged from [0.73–1.20] mm [17] and 0.69 mm [20]. Larger errors were found at the apical and basal locations, while better performance was obtained for the mid-ventricular LV portions. Furthermore, the analysis of signed errors showed no bias in the synthetic datasets. The tracking errors for both displacements and strains (Figs. 7 and 8) were moderately affected by the SNR values. In both cases, the median error values quickly converged to values that were only loosely dependent on SNR, with the largest errors observed at an SNR of 5. This stability is attributed to the use of a UNet-based architecture, where the convolution kernels and the contraction branch act as effective noise filters.

For the *external validation* dataset, error metrics on displacements showed median errors better than the MRI resolution. These errors are also in the range of those presented in the benchmark publication by [17]. For the in-vivo porcine dataset, predicted strain values were within the range of values reported in [36]. The predictions of radial, circumferential, and longitudinal deformations matched well with those of MA. Radial strain errors showed larger interquartile ranges compared to circumferential and longitudinal strains. These results are consistent with findings in [37], which showed that strain estimation, particularly radial strain, is influenced by tag-to-pixel size ratios. Similar conclusions were drawn in [17], [38], where significant underestimation of radial strains was observed. It was suggested that current protocols for 3D tagged MRI might not be optimal for the accurate quantification of radial strains. Additionally, anatomical segmentation and mesh extraction from the ED cine images were done as described in [35] and segmentation inaccuracies and slice misalignment could have contributed to inaccuracies in strain calculations.

## 6 Limitations

Although inference errors on the in-vivo datasets showed higher variability, than in the synthetic dataset, the results support nevertheless the use of synthetic images for network training. As noted in [20], in-vivo data could be used to fine-tune the network architecture and improve predictions on real data. Although time consuming, and potentially inaccurate for 3D tagged MRI [21], such fine tuning could compensate for the difference between synthetic and in-vivo datasets and reduce errors at inference time.

The full analysis of one case, presented in Fig. 11, took approximately 10 s per cardiac phase for displacement inference, mesh tracking, and strain calculation. This represents a significant speed-up compared to approaches presented in [17]. Results in Fig. 11b also demonstrate the physiological behavior of strains throughout the cardiac cycle.

## 7 Conclusion

We have developed a method based on synthetic MR images to train a neural-network-based approach for the automatic analysis of 3D tagged MRI without user intervention. The availability of a biophysical ground truth for all synthetic images allowed the training of the networks and their deployment on in-vivo data. Inference of displacements and strains on the full anatomy showed good agreement with ground-truth values and expected values from literature. Error metrics were comparable with the state-of-the-art on 3D tagged MRI data but with a net speed advantage as compared to previously proposed approaches.

## Author contributions

**Christian T. Stoeck:** Writing – review & editing, Investigation, Data curation. **Sebastian Kozerke:** Writing – review & editing, Resources, Project administration, Funding acquisition. **Stefano Buoso:** Writing – review & editing, Writing – original draft, Validation, Software, Project administration, Methodology, Investigation, Formal analysis, Conceptualization.

## Declaration of competing interests

The authors declare that they have no competing financial interests or personal relationships that could have influenced the work reported in this paper.

## Data Availability

The networks and the processing pipeline used in this work are publicly available at https://gitlab.ethz.ch/ibt-cmr-public/3dtagnet under MIT license conditions.
